# Editorial: Mechanisms of traumatic brain injury and its pharmacotherapy

**DOI:** 10.3389/fphar.2026.1854087

**Published:** 2026-05-14

**Authors:** Fernanda Guilhaume-Correa, Rebekah Mannix

**Affiliations:** 1 Division of Emergency Medicine, Boston Children’s Hospital, MA, United States; 2 Department of Pediatrics, Harvard Medical School, Boston, MA, United States

**Keywords:** brain injury mechanisms, neurotrauma, pharmacotherapy, TBI, traumatic brain injury, translational research

Despite the global public health burden of traumatic brain injury (TBI), effective pharmacological therapies remain elusive. This reflects, in part, the biological complexity of the injury itself. Primary mechanical damage initiates a cascade of secondary events that unfold across molecular, cellular, and systemic scales. TBI represents a dynamic, evolving process that is difficult to capture in any single experimental model and harder still to interrupt with any single intervention. Understanding and modulating this cascade is the central challenge, and the central ambition, of the work assembled here.

Taken together, the contributions in this Research Topic reframe TBI as a network disorder, in which interconnected molecular and cellular processes drive injury far beyond the initial insult. Among these, oxidative stress, neuroinflammation, and regulated forms of cell death emerge as central and mutually reinforcing mechanisms. Rather than acting in isolation, these processes form a tightly coupled system that amplifies tissue damage and constrains recovery.

Among the mechanisms explored in this Research Topic, ferroptosis emerges as a particularly compelling target. This iron-dependent, lipid peroxidation-driven form of regulated cell death sits at the intersection of metabolic dysfunction and oxidative injury—making it both mechanistically central and therapeutically tractable. Yang et al. demonstrate that the flavonoid fisetin attenuates ferroptosis through activation of the PI3K/AKT/NRF2 pathway, reducing reactive oxygen species and lipid peroxidation, restoring antioxidant defenses, and modulating ferroptosis-related proteins. Validated in a repetitive mild closed head injury mouse model and in HT22 neuronal cells, these effects translated into improved cell viability, neuronal survival, and functional outcomes, underscoring ferroptosis as a credible axis for pharmacological intervention after TBI.

Complementing this, additional studies expand the mechanistic landscape by examining lipid-mediated inflammatory signaling and its pharmacological modulation (Yang et al.; Sandler et al.; Xu et al.). Targeting the arachidonic acid–5-lipoxygenase–leukotriene axis emerges as a promising approach to reduce neuroinflammation, with pharmacological inhibition showing neuroprotective effects in TBI animal models (Yang et al.). Related work further supports the importance of inflammatory cascades and oxidative pathways as drivers of secondary injury (Sandler et al.; Xu et al.). Together, these findings reinforce the view that inflammation is not merely a byproduct of injury, but an active and targetable component of TBI progression.

The clinical contributions here remind us that some of the most informative signals in TBI remain physiologic. Eric Nyam et al. demonstrate that pupillary reactivity, a bedside marker of brainstem integrity, and age are independently associated with mortality in patients presenting with a GCS of 3. As a physiologic biomarker, pupillary response is notable precisely because it is dynamic, non-invasive, and mechanistically anchored, reflecting real-time neural function rather than a static snapshot. These findings also reinforce a broader theme for the field: advancing TBI therapeutics will require not only molecular targets but validated, clinically accessible biomarkers capable of stratifying patients and tracking treatment response across the injury timeline, well beyond the acute phase in which most TBI interventions are studied. Indeed, Elkinbard et al. highlight that TBI initiates neurological and systemic consequences that persist and evolve long after the primary injury, including post-traumatic epilepsy and sex hormone dysregulation with downstream effects on sleep and seizure susceptibility. Together, these contributions demonstrate that effective intervention in TBI cannot be confined to the first hours or days but must account for the chronic, progressive dimensions of a condition that continues to reshape neural and systemic function over time.

It is because of this mechanistic complexity and extended injury trajectory that the emphasis on multi-target pharmacological strategies across this Research Topic becomes particularly compelling. Natural compounds and bioactive molecules with pleiotropic effects are increasingly recognized for their ability to simultaneously modulate oxidative stress, inflammation, and cell death pathways. In this context, one study (Yan et al.) demonstrates that α-asarone significantly alleviates neuronal injury by enhancing autophagic flux through the miR-499-5p/PDCD4/ATG5 signaling pathway, leading to reduced cellular damage and improved neurological outcomes (Yan et al.). At the same time, drug repositioning offers a practical route to clinical application, using established safety profiles to investigate new neuroprotective effects. In support of this approach, a retrospective propensity score–matched analysis (Zhang et al.) reports that β-blocker administration is associated with improved survival and clinical outcomes in patients with TBI (Zhang et al.), highlighting the potential benefit of attenuating the catecholamine-driven stress response following injury (Zhang et al.). Collectively, these findings reinforce the concept that targeting interconnected pathological pathways and integrating both experimental and clinical strategies may be essential for improving outcomes in a condition driven by multiple overlapping mechanisms, such as TBI.

Together, these contributions combine to suggest a fundamental reconceptualization of TBI itself. Secondary injury emerges as a self-amplifying network, in which oxidative stress drives ferroptosis, ferroptosis fuels inflammation, and inflammation in turn sustains the conditions for further oxidative damage. Vascular dysfunction, metabolic failure, and immune activation are not parallel processes but intersecting ones with bidirectional effects. The implication is significant: interventions designed around single pathways, or single timepoints, are unlikely to reverse multisystem events.

Therapeutic progress also depends on increased fidelity of preclinical models. Despite decades of promising experimental findings, effective pharmacological therapies for TBI remain elusive, a gap that reflects translational noise from heterogeneous injury models, inconsistent intervention timing, and outcome measures that poorly mirror clinical endpoints. Two priorities stand out: 1) the definition of therapeutic windows; 2) the identification of biomarkers capable of tracking injury progression and treatment response in real time.

The work assembled here clarifies the direction of progress, which requires better translational frameworks rather than more mechanistic targets. The field has the mechanistic depth. What it needs now is the infrastructure to bring it to the bedside.

